# Knowledge Graph and Deep Learning-based Text-to-GraphQL Model for Intelligent Medical Consultation Chatbot

**DOI:** 10.1007/s10796-022-10295-0

**Published:** 2022-07-06

**Authors:** Pin Ni, Ramin Okhrati, Steven Guan, Victor Chang

**Affiliations:** 1 Institute of Finance and Technology, University College London, London, UK; 2 Department of Computer Science and Software Engineering, Xi’an Jiaotong-Liverpool University, Suzhou, China; 3 Department of Operations and Information Management, Aston Business School, Aston University, Birmingham, UK

**Keywords:** Text-to-GraphQL, Semantic parsing, Knowledge graph, Natural language processing, Deep learning, Health informatics

## Abstract

Text-to-GraphQL (Text2GraphQL) is a task that converts the user's questions into Graph + QL (Query Language) when a graph database is given. That is a task of semantic parsing that transforms natural language problems into logical expressions, which will bring more efficient direct communication between humans and machines. The existing related work mainly focuses on Text-to-SQL tasks, and there is no available semantic parsing method and data set for the graph database. In order to fill the gaps in this field to serve the medical Human–Robot Interactions (HRI) better, we propose this task and a pipeline solution for the Text2GraphQL task. This solution uses the Adapter pre-trained by “the linking of GraphQL schemas and the corresponding utterances” as an external knowledge introduction plug-in. By inserting the Adapter into the language model, the mapping between logical language and natural language can be introduced faster and more directly to better realize the end-to-end human–machine language translation task. In the study, the proposed Text2GraphQL task model is mainly constructed based on an improved pipeline composed of a Language Model, Pre-trained Adapter plug-in, and Pointer Network. This enables the model to copy objects' tokens from utterances, generate corresponding GraphQL statements for graph database retrieval, and builds an adjustment mechanism to improve the final output. And the experiments have proved that our proposed method has certain competitiveness on the counterpart datasets (Spider, ATIS, GeoQuery, and 39.net) converted from the Text2SQL task, and the proposed method is also practical in medical scenarios.

## Introduction

The current medical question-and-answer system has been widely used in many fields, especially in healthcare scenarios such as hospital guidance and reception and online medical consultation. Therefore, creating a more robust medical dialogue system can undoubtedly make greater contributions to a more advanced integrated intelligent medical system. The current medical dialogue system at the business level is generally implemented through rule-based question and answer templates. That is, this requires a large number of professional question and answer scene templates to be preset and then match similar questions and answers through retrieval. However, the current more advanced neural network-based pipeline model replaces the previous simple matching model and instead uses a complex deep learning architecture to perform more complex text representation, retrieval, and matching tasks. As an emerging dialogue system paradigm, this approach reduces the drawbacks of the semantic analysis of the regular expression template method implemented in the form of symbol matching and provides a new breakthrough opportunity for technological development in this field. At the same time, the knowledge graph provides the intelligent dialogue system with a continuously updated external knowledge source and creates a more flexible and extensive expansion for the natural language question and answer system. Therefore, by integrating the continuously expanding knowledge graph and neural networks-based components with better performance in each subtask, the performance of the dialogue system in terms of domain adaptability, recognition accuracy, and interaction quality can be improved from the level of the various modules.

The study mainly proposed an encoder-decoder pipeline composed of XLM with the introduction of the “Schema-Utterance” knowledge mechanism and Point Network. Among them, XLM learns richer Text2GraphQL knowledge by inserting a pre-trained “Adapter” (a plug-in that links GraphQL Schemas and the corresponding utterance for pre-training) to use it as an encoder for the model. The Pointer Network as a decoder calculates the context vector by combining the Attention mechanism and restricts them through the guide mechanism. And decides the token output at each step according to the weight distribution as well as improved by an adjuster mechanism. Finally forms a statement sequence that is translated into GraphQL.

This study also used the text data of a large online medical Q&A forum to construct a medical Q&A knowledge graph. The Q&A dataset annotated in this knowledge graph and the derived GraphQL format verifies the effect of the proposed model. At the same time, this work is the first Text-to-GraphQL work, and there is currently no dedicated public dataset and graph database available. Therefore, we also use the existing Text-to-SQL evaluation dataset to convert to Text-to-GraphQL format for the effective verification of the proposed model. This method has also obtained competitive performance on mainstream Text2SQL datasets such as Spider 1.0, ATIS and GeoQuery.

Section 1 introduces the overall background of the task of Text2GraphQL. Section 2 focuses on sorting out the progress of previous similar works with methods or tasks, etc. Section 3 introduces the components of the entire pipeline. Section 4 details the experiment. Section 5 is about results and analysis; Sect. 6 is conclusions. Section 7 is limitations and future work.

## Related Works

### Text-to-SQL Task

The medical dialogue system is mainly used to meet the interactive needs in complex medical scenarios, mainly by translating the natural language that carries human thinking into specific operating instructions. This is a huge comprehensive system composed of a variety of complex rules-based or deep learning-based natural language processing components. And Text-to-SQL is a branch of NLP research and is dedicated to automatically translating human needs described by natural language into a language that machines can understand for more flexible and convenient queries or interactive actions. This field is an emerging research direction, and the previous mainstream work is presented as follows:

TypeSQSL is a knowledge-based type-aware text-to-SQL generator proposed by Yu et al. (2018a). The purpose is to better understand the semantics and recognize rare entities and numbers in utterance questions by converting Text2SQL tasks into slot filling tasks and using type information. Compared with SQLNet (Xu et al., 2017) and Seq2SQL on the WikiSQL dataset (Zhong et al., 2017), their model has lower time consumption and better performance. In addition, currently, only a single data set is used in the training and testing process, and there is a small number of logical forms or annotation labels. And the content of the existing data set is relatively simple, containing only simple SQL queries and single tables (e.g., WikiSQL), which cannot test the model's generalization ability in new fields and the true semantic parsing performance on unknown complex programs. Therefore, Yu et al. (2018c) built a database (Spider) covering more than 5,693 complex SQL queries, 10,181 problems and 200 tables. It also defines a new semantic parsing task. The models must be fed questions and database schemas to forecast previously unknown queries on the new database. This work that uses the dataset as a benchmark for model evaluation also includes SyntaxSQLNet (Yu et al., 2018b), RCSQL (Lee, 2019), and GNN for Text-to-SQL Parsing (Bogin et al., 2019).

SyntaxSQLNet (Yu et al., 2018b) is the first model designed for Spider tasks. In decoder processing, instead of generating linear text, it generates a syntax tree corresponding to the characteristics of the SQL language. And the paper proposes a method to generate cross-domain training data, using data enhancement to improve model performance. RCSQL (Lee, 2019) mainly includes building decoders for different SQL statements, using recursive methods to generate sub-queries; using Seq2Seq instead of seq2set for column name prediction, etc. GNN for Text-to-SQL Parsing (Bogin et al., 2019) is mainly to express the data structure of the relational database with neural networks to improve the utilization efficiency of database information. And the GNN structure is used to assist the subsequent encoding and decoding process. The work of IRNet (Guo et al., 2019) can be divided into three stages. First, schema linking the question and the relational structure of the database. Then, construct a SemQL query using a grammar-based neural network. Finally, construct SQL queries in different scenarios based on SemQL. To establish a comprehensive framework for schema encoding and schema linking, RAT-SQL (Wang et al., 2020) employs a relation-aware-based self-attention mechanism. Simultaneously, more edges are defined on the directed graph, and the schema is further decomposed.

### Rule-based and Deep Learning-based Dialog Systems

The rule-based question answering system is more interpretable and controllable, and cold start is easier. In the case of no data or very little data, the rule-based method can be used to launch a rudimentary question and answer system quickly. But it does not understand semantics but is based on symbolic matching. In other words, with the rule-based method, it is necessary for the designer of the rule to consider all the circumstances as much as possible. However, this is very difficult, which does not include the problems of synonyms and sentence structure. Many previous studies have proved that (Cui et al., 2020; Hong et al., 2020; Lee et al., 2019), through the end-to-end model as a component of different tasks, will play a greater role in the overall system, especially in text feature modeling. And many previous studies are deep learning-based pipelines.

Li et al. (2020c) proposed a neural network model to generate diverse coherent responses on the basis of TransferTransfo (Wolf et al., 2019). Their model mainly uses intent and semantic slots to represent intermediate sentences to guide the generation process. They also design a filter to select appropriate dialogue responses.

Chuan and Morgan (2020) collected clinical trial eligibility criteria from official channels to evaluate chatbots and classifiers and proved that the performance of active deep learning classifiers is better than the baseline K-Nearest neighbor method. And through the construction of chatbots to evaluate participants' understanding of eligibility, as well as the rating of chatbot interface in terms of interactivity, perceived usability and dialogue.

Abd-Alrazaq et al. (2019) reviewed 41 unique chatbots that can be used for mental health in the past 1,039 articles. These chatbots mainly focus on autism and depression and are mostly used in aspects such as screening, training and therapy. Most of these chatbots are rule-based and implemented in stand-alone software.

Kandpal et al. (2020) also discussed the working principles of various dialog system frameworks and related works and applications in this field in their work, as well as the challenges and scope of related technologies.

Lai et al. (2020) proposed a simple dialogue state tracking model based on BERT. The model can run when the domain ontology may change dynamically, and the number of parameters will not increase as the size of the ontology increases. In addition, their model has achieved better results on the WoZ 2.0 dataset (Wen et al., 2017) than the previous methods (Chao & Lane, 2019; Mrkšić et al., 2017; Nouri & Hosseini-Asl, 2018; Ren et al., 2018; Zhong et al., 2018).

In addition, there is a lot of work on rule-based or deep learning-based chatbots or other NLP human–computer interaction components (Khilji et al., 2020; Ni et al., 2020c; Reddy et al., 2020) and have provided many contributions to the development of this field. This includes a large number of novel NLP components, or related methods (Amith et al., 2019; Dai et al., 2019; Ni et al., 2019, 2021a, 2021b), which can also provide benefits for this area.

### Rule-based NLP and Semantic Parsing Task

In the early stages of natural language processing, most Rule-based NLP methods are based on Linguistic rules and patterns for semantic parsing (Polanyi et al., 2004). This commonly used traditional method mainly realizes specific natural language tasks according to preset rules or templates. The advantage of this method is that the established rules can be used to match the specific content existing in the text precisely. Its precise and fast matching capability, efficient cold start capability, and controllable result output capability make it one of the best practices in the industry. The early rule-based methods strongly relied on the feature extraction rules and templates formulated by experts.

Vilares et al. (2017) evaluate the accuracy of task-oriented syntactic parsing to see how the accuracy of parsing affects the performance of current SOTA (state-of-the-art) rule-based sentiment analysis systems. In the study, they also pointed out that parsing is a computationally expensive task, and it would be wiser to prioritize speed over accuracy. This is also the advantage of the rule-based approach.

Ramasamy & Žabokrtskỳ (2011) conducted a comparative experiment through the rule-based method of Dependency Treebank and the method based on a corpus, the two dependency parsing methods. Finally, it is found that corpus-based methods have greater advantages over unlabeled data.

Gotab et al. (2009) proposed an active learning schema based on the Spoken Language Understanding (SLU) criterion, a criterion for automatically updating SLU models for deployed speech dialogue systems. This work compares two SLU models, rule-based and corpus-based. The rule-based model is composed of thousands of manual rules for system deployment. In contrast, the corpus-based model is based on classifiers that are automatically learned on the annotated corpus. This also verifies that the rule-based method is more suitable for efficiency-oriented application scenarios that require fast loading.

### Corpus-based NLP and Semantic Parsing Task

Unlike rule-based methods, corpus-based methods can automatically learn potential rules from a larger amount of data without the need to formulate rules manually. This is a data-driven approach, where the corpus is often drawn from massive amounts of data to discover underlying patterns and adapt iteratively. Therefore, this type of approach is more suitable for situations with rich data sources, whether they are annotated or not. Typical representatives include pre-trained language models that can perform a variety of NLP practical tasks.

The pre-trained language model (PLM) is driven by a large amount of corpus and can use these data to realize the semantic representation of knowledge contained in a large amount of text to realize downstream tasks. The downstream tasks include natural language processing tasks such as classification (Li et al., 2019b; Maltoudoglou et al., 2022; Ni et al., 2020a, 2020b), sequence labeling (Dai et al., 2019; Li et al., 2020b), summarization (Chintagunta et al., 2021; Lacson et al., 2006; Yuan et al., 2021), translation (Névéol et al., 2018; Nobel et al., 2021; Wang et al., 2019), generation (Melamud & Shivade, 2019; Peng et al., 2019; Xiong et al., 2019), etc. As one of the new downstream tasks, the translation task, Zhu et al. (2020) previously found that using the pre-trained language model as contextual embedding instead of direct fine-tuning will produce better results. Based on this, they propose a method to extract the representation of the input sequence using PLM, and then fuse the representation with each layer of the encoder and decoder of a neural machine translation (NMT) model through an attention mechanism. This study is a typical method for transforming sequences by using BERT as a feature extraction part and combining NMT. Similar ones include He et al. (He & Choi, 2020) using PLM as a context representation layer to combine Biaffine parser to realize semantic parsing tasks, etc. Experiments on datasets from SemEval 2015 Task 18 (Oepen et al., 2015) and SemEval 2016 Task 9 (Che et al., 2016) also demonstrate the effectiveness of the corpus-based approach. Therefore, corpus-driven NLP methods have certain advantages in semantic parsing tasks.

## Methodology

As a practical task that has not been explored, Text-to-GraphQL can be regarded as a sub-research direction of semantic analysis. It is different from Text-to-SQL in that it is oriented to graph databases and graph query languages. Compared with traditional relational databases, graph databases are more flexible in terms of graph structure data suitable for complex medical relationship networks (e.g., directly traversed on the graph). This makes more and more knowledge bases appear in the form of graph structures. The graph database is directly accessed as a class pointer, and it also has a more efficient operation of linking data than a relational database. And due to the continuous update and iteration of external data, the content and format of the data will continue to change. For relational databases, this means that the structure and number of tables need to be constantly changed, which has a greater impact on changes in source data. For the graph database, only vertices, edges, and attributes need to be added, updated and set to the corresponding type. Therefore, in contrast, the graph database pays more attention to the individual data and the relationship between them and is also more suitable as a medical knowledge base for continuous expansion to support the construction of a more intelligent medical dialogue system. Unfortunately, there is currently a lack of a semantic parsing solution for graph query languages.

The medical question answering system mainly includes the following parts: 1. Data source (medical text information source), 2. Knowledge extraction layer (e.g., Named Entity Recognition, Regular Expression Matching), 3. Knowledge storage layer (e.g., graph database), 4. Knowledge application layer (e.g., Q&A system). The data source mainly comes from the online medical encyclopedia (e.g., 39 Health). The knowledge extraction layer is an extraction mode that combines Regular Expression Matching and deep learning-based named entity recognition models. We choose Neo4j as the graph database for storing medical knowledge data in the knowledge storage layer. The knowledge application layer is mainly a question-and-answer system built based on the previous three layers. The system is mainly composed of rule-based template matching and deep learning-based Text-to-GraphQL. And to realize the translation of the natural language input by the user into a graph database query and respond suitably.

The method we propose mainly focuses on how to translate natural language into graph database queries. This method needs to convert text queries into graph retrievals to match appropriate responses. The current Text-to-SQL task is also analogous to neural machine translation in that it is a sequence-to-sequence (Seq2Seq) generation task. This type of Seq2Seq model that introduces mechanisms such as Attention can generally achieve an accuracy of about 80% on multiple single-domain data sets, but it is generally less than 25% on multi-domain data sets. However, limited by the strict logical structure of the database query language, it is necessary to ensure rationality and executable grammar. Therefore, the standard Seq2Seq framework is unable to model this information.

The conventional Seq2Seq paradigm cannot address the issue that the vocabulary of the output sequence changes as the length of the input sequence changes. In some tasks, the output is strictly dependent on the input, or the output can only be selected from the input. For example, enter a paragraph and extract the most critical words in the sentence. Or input a string of numbers and output related semantic queries around these numbers. At this time, if the traditional Seq2Seq model is used, it is ignored that the output can only choose this prior information from the input. Pointer Network is proposed to solve this problem.

Pointer Network is mainly used to solve combinatorial optimization problems (TSP, etc.) (Golden et al., 1980) and others (e.g., Convex Hull (Van Rooij et al., 2003)), which is essentially an extension of the encoder/decoder RNN of the Seq2Seq type. Mainly used to solve the problem that the output dictionary length is not fixed. In a combinatorial optimization problem such as TSP, the coordinate sequence of the input city is also the coordinate sequence of the city output, and the city scale $n$ calculated each time is also not fixed. The output of each decoder is actually the probability vector of a city currently selected, and its dimension is $n$, which is the same length as the sequence vector input by the encoder. Based on this concept, we generalize it to Text2GraphQL, a task-oriented NLP and structured languages.

Since the conventional Seq2Seq model is a model including Encoder and Decoder, it mainly transforms one sequence into another sequence. However, since the predicted output target size of the Seq2Seq model is fixed, it is difficult to solve some situations where the output target size will change (e.g., combinatorial optimization problem). The number of output targets of a combinatorial optimization problem depends on the length of the input sequence. For example, a machine translation task contains $n$ characters (1, 2, 3… $n$), and the number of target output in another language is $n$. The Pointer Network (Vinyals et al., 2015) can solve the problem of variable output dictionary size. The output dictionary size is equal to the length of the Encoder input sequence and Attention is modified to make it suitable for combinatorial optimization problems. It can get the probability of each token in the input sequence according to Attention (i.e., the output is selected from the input).

The Decoder of the output of Seq2Seq predicts the output of each position (but the number of output targets is fixed). Pointer Network's Decoder directly obtains the probability of each position in the input sequence according to Attention and takes the input position with the highest probability as the current output.

The word list used in the decoder part of the conventional Seq2Seq model is fixed. In other words, it is selected from the fixed word list in the generated sequence. But Text-to-GraphQL is distinct from the general Seq2Seq task. It may appear in the generated sequence: a) words that appear in question sentences; b) traversal statements of GraphQL; c) corresponding vertices and edges in the database. These challenges are well solved by the Pointer Network, and the vocabulary employed in its output changes depending on the input. The specific method involves directly selecting words from the input sequence as output using the Attention mechanism.

In the Text-to-GraphQL task, it can consider the user’s question and other words that may appear in the target GraphQL statement as the input sequence (node/edge name sequence; GraphQL function list; question word sequence), using Pointer Network select words directly from the input sequence as output. At each step of the decoder, the Attention score is calculated with each hidden layer state of the encoder, and the maximum value is taken as the current output and the input of the next step. In addition, in the process of “translation”, proper nouns often appear, such as subjects or objects such as names of persons and places. Pointer Network can be used to directly extract the nouns corresponding to the original text and copy them directly into GraphQL for filling.

### Encoder Layer

The basic structure of the proposed model is composed of two parts: Encoder and Decoder. Therefore, in the Encoder part, we first use the XLM (a Transformer-based cross-lingual pre-trained language model) (Conneau & Lample, 2019) to encode natural language text. This part will learn semantic features and provide support for the subsequent conversion of the word embedding layer. The XLM is an embedding model for encoding utterance and the corresponding schema together in the training process. Here we fine-tune the XLM model by using $\left[CLS\right]$ and $\left[SEP\right]$, which are special tokens in the transformer, to separate natural language queries from GraphQL schemas. They are represented as follows.

**Fig. 1:**
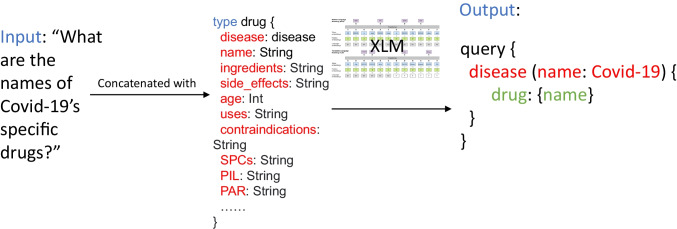
An example of input and output containing fields and arguments for GraphQL schema

$\left[CLS\right]{U}_{1},{U}_{2},{U}_{3},\dots ,{U}_{i}\left[SEP\right]{S}_{1},{S}_{2},{S}_{3},\dots ,{S}_{j}\left[SEP\right]$ (Hwang et al., 2019). Where ${U}_{i}$ is the $i$-th token or word in the utterance. ${s}_{j}$ is the corresponding $j$-th schema. As shown in Fig. 1, there is richer information in some specific schemas (e.g. *Covid-19*), which are usually specific fields or arguments in this type of schemas. These details will be further decoded and extended by the decoder.

#### Schema Learning Layer

**Fig. 2:**
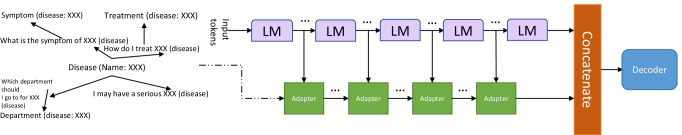
Inject the linking of GraphQL schemas and different utterances into the language model

**Fig. 3:**
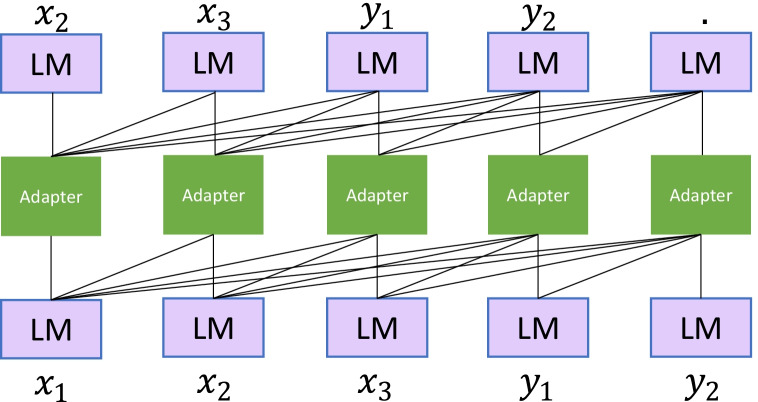
The plug-in “Adapter” in the language model

**Fig. 4:**
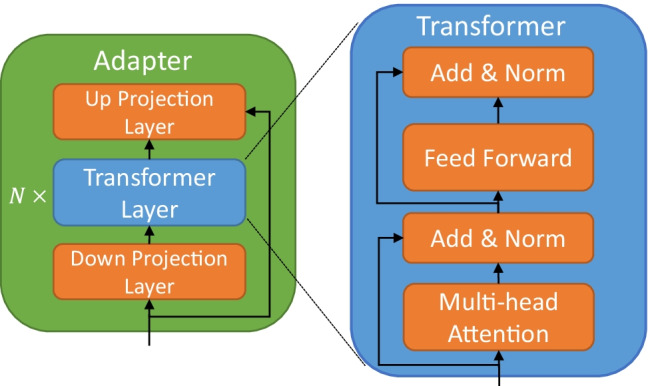
The structure of “Adapter” and “Transformer” and the relationship between them

This layer is a brand-new mechanism that “injects” the linking between the diversified prior knowledge of natural language expressions (utterances) and their corresponding logical form (GraphQL schemas) into the language model. They are packaged into “Adapter” modules, which are inserted into each step of the language model sequence as plug-ins (Figs. 2, 3). The Adapter allows the model to learn different forms of natural language expressions and contextual information corresponding to different types of schemas. Therefore, it can play a role equivalent to knowledge alignment, disambiguation, or coreference resolution in language models. Referring to the work proposed by Wang et al. (R. Wang et al., 2021), so the Adapter module is mainly composed of an up-projection layer, $N$ layer Transformer Encoder Layers, and a down projection layer (Fig. 4). By outputting each layer of the language model except the last layer, it is passed to the corresponding layer of the Adapter (i.e., Transformer layers of the $M$ layer, corresponding to the Adapter layers of the $K$ layer). In the case of a single Adapter, the features of the last layer of the language model are spliced with the features of the last layer of the Adapter and finally passed into a specific training task. In the case of multiple Adapters, the features of the last layer of the language model are spliced with the features of the last layer of Adapter 1 and Adapter 2, and then transferred to the training task.

**Fig. 5:**
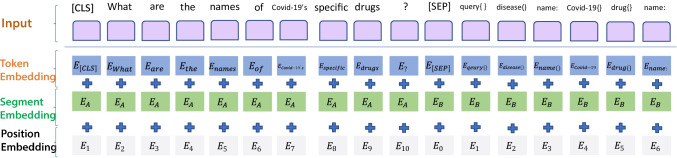
Putting Utterance and GraphQL schemas together for XLM pre-training

In this part, we mainly used the FacAdapter schema in Wang et al. (2021) to extend it to a training task. Specifically, it is transformed into a special relationship classification task. That is, a given context and a pair of textual entities, as well as the corresponding GraphQL statements and key schemas in them. And this multi-instance, multi-entity cross-relation classification task is mainly through the joint training of natural language expressions and GraphQL and their entities for classification. Therefore, this task can also be regarded as a cross-language relation extraction task (e.g., a single mention refers to multiple schemas in the GraphQL statement at the same time) (Fig. 5). This allows the language model to learn the relationship between GraphQL schemas and the corresponding wide range of expressions through this mechanism.

The FacAdapter is to implement external knowledge injection. The relational network “injected” here is based on the dataset used in the medical knowledge graph mentioned above, so the schema linking module is trained based on this dataset. The entities and relationships are extracted directly from the raw dataset, and the corresponding GraphQL schema annotations are added. This relationship extraction task is to use the annotated data to extract the GraphQL schemas involved in each utterance (or vice versa) and their relationships. This is because each GraphQL schema token has correspondence to multiple utterance expressions and is presented differently depending on the context, but their core meaning usually still points to a particular schema or schemas. Their specific schema also contains different fields and arguments that may be mentioned in the utterance, so sometimes, a single utterance sentence will point to multiple types of single or multiple schemas or fields/arguments therein at the same time. This allows the Adapter to learn the complex meanings and relationships in some utterances through these structured semantic networks, enabling the Adapter to help XLM learn more utterance expressions about each schema correspondence.

The FacAdapter model contains $k$ Adapters. Each Adapter layer contains $N$ transformer layers, two mapping layers and one residual connection. The Adapter layers are connected to the different transformer layers in XLM.

There, the input of the current Adapter layer contains 2 layers: 1. the hidden layer of the Transformer layer output, and 2. the output of the previous Adapter layer. These two representations need to be concatenated together.

The output of the Adapter model: 1. the output of the last hidden layer of XLM; 2. the output of the last Adapter layer. After contacting them, they will become the final output.

This part will improve the output of XLM to allow it to generate embedding for utterances that better match the schema.

### Decoder Layer

#### Pointer Network

**Fig. 6:**
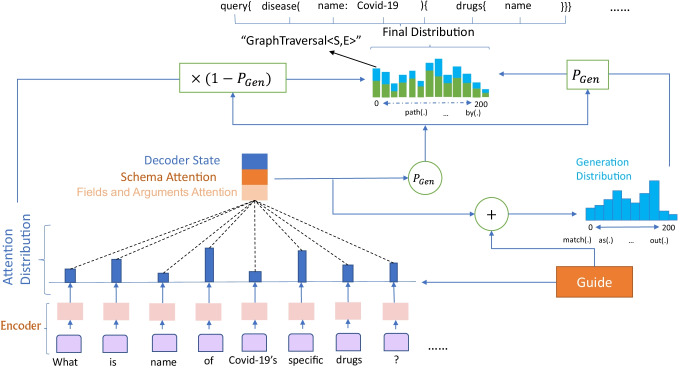
Decoder structure (Pointer Network)

After obtaining the input from the embedding, it is passed into the pointer network (Fig. 6) for further decoding to obtain the hidden vector ${h}_{i}$. For the decoder, a word embedding is received at each step along with a hidden layer representation of the previous unit ${s}_{t}$, which is the previous token of the target output during training and it is the previous token that the decoder emitted out during testing.

In this part, their weights or attention distribution ${a}^{token}$ can be calculated by the Attention mechanism (Bahdanau et al., 2014). It can also be regarded as the probability distribution of attention of all utterance tokens in the source input, which is used to tell the decoder which token should pay attention to generate the corresponding schema. Where ${W}_{s}$, ${W}_{h}$, ${v}^{T}$ and ${b}^{atten}$ are trainable parameters (Zhang et al., 2021).

1 $${z}_{i}^{token}={v}^{T} \mathrm{tanh}\left({W}_{s} {s}_{t}+{W}_{h} {h}_{i}+{b}^{attn}\right)$$

2 $${a}_{t}^{token}=Softmax\left({z}^{token}\right)$$

Attention distribution can also be seen as a distribution of the information needed for the current decoding step $t$ in the source input, which is used to generate a weighted sum of the encoder hidden states, i.e., the context vector ${c}_{t}^{token}$.

3 $${c}_{t}^{token}={\Sigma }_{i}{a}_{i}^{token}{h}_{i}$$

Inspired by the encoding and decoding methods for the “column head” in SQL in PG-GSQL (Wang et al., 2020), Editing-Based SQL (Zhang et al., 2019) and CD-Seq2Seq (Yu et al., 2019), our encoder part also incorporates a similar mechanism to enable utterances to find their potentially required matching schema extensions by computing the Schema Attention distribution. Thus, our pointer network also considers the fusion of 2 kinds of dimensional information: 1. GraphQL Schema Attention, 2. Fields and Arguments Attention.

1. GraphQL Schema Attention
  This part matches the encoder output with all the schemas in the “vocabulary” by computing the Attention distribution. The Attention distribution is used to determine which GraphQL Schemas are mentioned in the corresponding utterance.
2. Fields and Arguments Attention

After 1 is done, a finer-grained Attention distribution is calculated again. This is to extend the details in the GraphQL statement. This is to help all candidate GraphQL schemas find those required fields and arguments they contain.

These two parts of the Attention distribution, which we concatenate to obtain the context vector via $C$, are represented as follows:

4 $${c}_{t}=\left[{c}_{t}^{\text{schema}};{c}_{t}^{token}\right]$$

After that the attention vectors combined with the hidden vectors ${s}_{t}$ of the decoder, after two linear layer operations, the probability distribution over the vocabulary can be obtained as follows:

5 $${f}_{t}=V\left[{s}_{t};{c}_{t}^{token}\right]+b$$

6 $${f}_{t}^{\mathrm{^{\prime}}}={V}^{\mathrm{^{\prime}}}\cdot {f}_{t}+{b}^{\mathrm{^{\prime}}}$$

7 $${P}_{vocab}=Softmax\left({f}_{t}^{\mathrm{^{\prime}}}\right)$$

where ${V}^{^{\prime}}$, $V$, ${b}^{^{\prime}}$, $b$ are trainable parameters. The final probability of generating a certain token $k$ at the current decoding step is:

8 $$P\left(k\right)={P}_{vocab}\left(k\right)$$

The loss at each time step $t$ is the negative log-likelihood of the current target token. And uses cross-entropy to calculate the loss, the full loss is the average of the loss at each position in the sequence:

9 $$loss=\frac{1}{T}{\sum }_{t=0}^{T}\left(-{\Sigma }_{t}\mathrm{log}P\left({k}_{t}\right)\right)$$

### Pointer Generator

The Pointer Network mechanism solves the problem of filling the “argument/value” (i.e., out-of-vocabulary, OOV) in a GraphQL statement with the objects mentioned in utterance. The model can generate the corresponding token with a certain probability (${p}_{gen}$) and copy the required “argument” or “value” of objects from the source input with a certain probability (${1-p}_{gen}$). Depending on the size of ${p}_{gen}$, it is allowed to generate tokens and copy “arguments/values” as well. The “OOV” problem can be solved by combining both extraction and generation methods.

${p}_{gen}$ is generated by the attention vector ${h}_{t}$, the hidden vector ${s}_{t}$, and the embedding vector ${x}_{t}$ of the current decoding step $t$. This is to copy the token from the utterance input sequence by sampling from the attention distribution ${a}^{token}$, and generate the corresponding schema from the vocabulary by sampling from ${P}_{vocab}$.

$\upsigma$ is the sigmoid function, scalar ${b}_{\text{ptr}}$ and vectors ${k}_{s}$, ${k}_{x}$, ${k}_{c}$ are learnable parameters. It can be specifically formalized as:

10 $${p}_{gen}=\upsigma \left({k}_{s}^{T}{s}_{t}+{k}_{c}^{T}{c}_{t}+{k}_{x}{x}_{t}+{b}_{\text{ptr}}\right)$$

For a given token $k$, its generation probability is:

11 $$p\left(k\right)=\left(1-{p}_{gen}\right){\Sigma }_{i:{k}_{i}=k}{a}_{i}^{token}+{p}_{gen}{P}_{vocab}\left(k\right)$$

Here the copy probability is obtained by summing the attention distribution, ${\Sigma }_{i:{k}_{i}=k}{a}_{i}^{t}$ denotes that for the word $k$, its copy probability is the sum of the attention distributions of the words $k$ in utterance.

The main input of this model is natural language text and logical form expression (i.e., GraphQL Statement). Our method is mainly to input natural language into the model and converts it into GraphQL statements, and according to the semantics learned from natural language sentences, output the corresponding GraphQL tokens step by step, and finally form a complete GraphQL statement. Therefore, the main structure of the Pointer Network is that XLM is used as the Encoder Hidden States to encode the original text into a hidden state, and then BiGRU is used as the guide layer to restrict the corresponding GraphQL tokens output within a certain range.

#### Pointer Network Output

Compared to other traditional Seq2Seq models, there is no direct output from the Decoder Layer in our Pointer Network method. Instead, a context vector (mainly in the form of a vector distribution) is formed by the encoder input after the Attention calculation and then integrated with the output of the Decoder layer into a soft decision mechanism ${P}_{copy}$. The decision-making mechanism can be regarded as a probability filter, mainly to determine whether the current prediction is to directly copy a token (usually an argument or value of the object name) from the source input utterance or generates a token from the GraphQL function list.

#### Guide Component

We add a Guide component to the pointer network to restrict the predicted GraphQL schema category to a reasonable range.

Learning the category list of schemas in the GraphQL dataset during the training phase. and by classifying the input utterance in the encoder with a reasonable type. This is to make predictions about objects and their fields within a reasonable category range to reduce dependency errors for arguments or variables pointed to by objects. The category in this context refers to a specific set of objects of a particular category. For example, the “drug” category is the set of all objects about “drug”, which is different from the set of objects under the category “treatment”. The content of the objects collection is different, but there may be records that point to each other in the arguments under their respective fields. Therefore, a category is similar to a table in SQL and usually, objects under the same category have similar fields because their attributes are usually similar (similar to row in SQL representing each record). Determining in which GraphQL schema category they should be classified is further processed in a reasonable way.

In this component, we pre-classify them by transformer and softmax.

#### Adjuster Component

The overall end-to-end network structure is also supported by the Adjuster component. The Adjuster integrates the hierarchical copy-based pointer architecture with the Transformer, which ultimately enables the training of the pointer network with the target output samples as the generation target to adjust the output of the pointer network. This includes the task of identifying the bounds of argument values (e.g., the term “Covid-19” in the sample as a custom argument, which does not exist in the GraphQL schema list, i.e., “vocabulary”). This should be copied from the target output to adjust the probability distribution of the output token in the Pointer Network.

Therefore, during training, the target output (i.e., GraphQL statement), as the expected output of supervised learning, is first transformed into the decoder state $d$ by the Transformer after obtaining its embeddings. Which first expresses the candidate representations of the $i$-th embedding by ${e}_{i}=\left\{{e}_{i}^{\left(1\right)},\dots ,{e}_{i}^{\left(k\right)}\right\}$. In addition to this, we also improve it with Self-Attention before interacting with the encoder states of the Transformer to better capture their semantics. Here, both Layer normalization (LN) (Ba et al., 2016) and residual connection (He et al., 2016) are also used in both sub-layers of the encoder and decoder.

12 $$\stackrel{\sim }{{e}_{i}^{\mathrm{^{\prime}}}}=SelfAtt\left(Emb\left({m}_{i}\right)\right)+Emb\left({m}_{i}\right)$$

13 $$\stackrel{\sim }{{e}_{i}}=LN\left(\stackrel{\sim }{{e}_{i}^{\mathrm{^{\prime}}}}\right)$$

14 $${e}_{i}^{\mathrm{^{\prime}}}={{FFN}}\left(\stackrel{\sim }{{e}_{i}}\right)+\stackrel{\sim }{{e}_{i}}$$

15 $${e}_{i}=LN\left({e}_{i}^{\mathrm{^{\prime}}}\right)$$

The embedding of the Target Output is based on the Transformer. Therefore, this Encoder is made up of $N$ identical layers, each of which has two sub-layers: SelfAtt (Self-Attention) and FFN (Fully Connected Feedforward Network), in that order. ${o}^{l}=\left\{{o}_{1}^{l},{o}_{2}^{l}\dots ,{o}_{n}^{l}\right\}$ denotes the output of the $l$-th layer, and ${o}^{N}$ is the representation of the encoding state of the last encoder layer.

16 $$\stackrel{\sim }{{o}^{{\mathrm{^{\prime}}}{l}}}=SelfAtt\left({o}^{l-1}\right)+{o}^{l-1}$$

17 $${o}^{{\mathrm{^{\prime}}}{l}}={{FFN}}\left(\stackrel{\sim }{{o}^{l}}\right)+\stackrel{\sim }{{o}^{l}}$$

18 $$\stackrel{\sim }{{o}^{l}}=LN\left(\stackrel{\sim }{{o}^{\mathrm{^{\prime}}l}}\right); {o}^{l}=LN\left({o}^{\mathrm{^{\prime}}l}\right)$$

For the Transformer Decoder, the structure is similar to the raw Transformer Encoder but with the addition of a Cross-Attention (CroXAtt) layer for information capture on the encoder part. Similarly, ${d}^{l}$ is the output of the $l$-th decoder layer, while ${d}^{N}$ is the output of the last decoder state $d$.

19 $$\stackrel{\sim }{{d}^{\mathrm{^{\prime}}l}}=SelfAtt\left({d}^{l-1}\right)+{d}^{l-1}$$

20 $$\widehat{{d}^{\mathrm{^{\prime}}l}}=CrossAtt\left(\stackrel{\sim }{{d}^{l}},h,h\right)+\stackrel{\sim }{{d}^{l}}$$

21 $${d}^{\mathrm{^{\prime}}l}=FFN\left(\widehat{{d}^{l}}\right)+\widehat{{d}^{l}}$$

22 $$\stackrel{\sim }{{d}^{l}}=LN\left(\stackrel{\sim }{{d}^{\mathrm{^{\prime}}l}}\right); \widehat{{d}^{l}}=LN\left(\widehat{{d}^{\mathrm{^{\prime}}l}}\right);{d}^{l}=LN\left({d}^{\mathrm{^{\prime}}l}\right)$$

Finally, the probability $p\left( {y_{t} \left| {y < t,x} \right.} \right)$ of the $t$-th target GraphQL token is output by linear and softmax. $y<t$ is the proceeding tokens before ${y}_{t}$. $x=\left\{{x}_{1},{x}_{2},.,{x}_{n}\right\}$ represents the source sequence of target output.

23 $$p\left( {y_{t} \left| {y < t,x} \right.} \right) \propto \exp \left( {W_{o} d_{t} } \right)$$

**Fig. 7:**
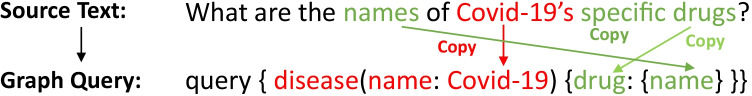
Copy of arguments/values of objects in the “Translation” process of Pointer Network

**Fig. 8:**
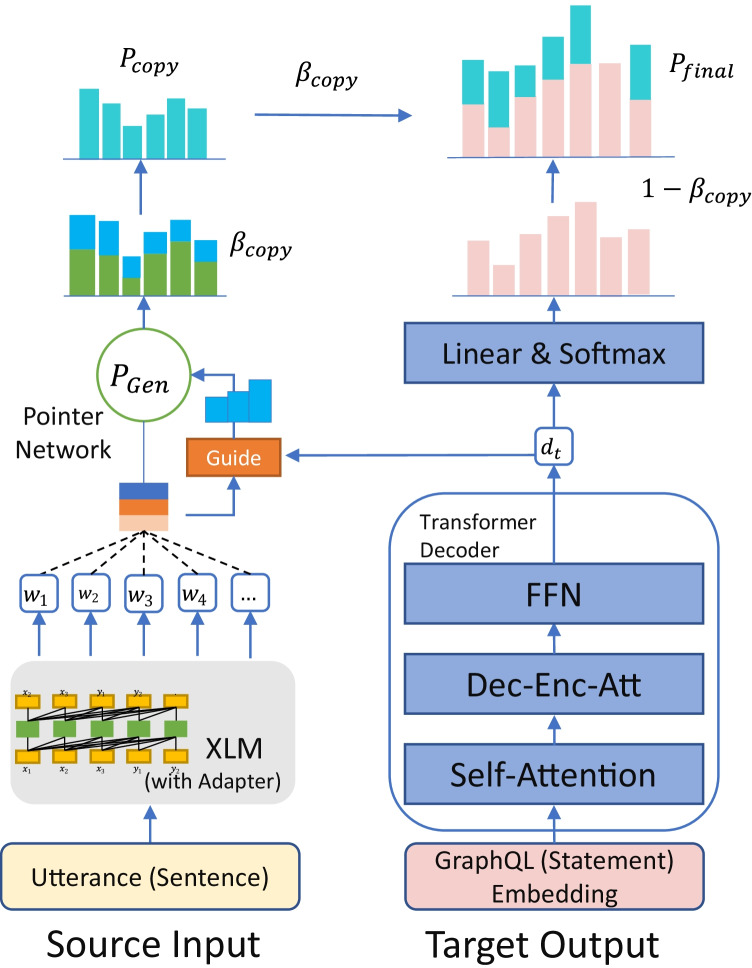
Adjuster: References and corrections during training

Then, we need to weighted concat the probability distribution of the final Transformer's decoder output token with the probability distribution of the token generated by the pointer network part. This is used to adjust the GraphQL statement output by the pointer network (a complete sequence of logical forms including operation name, objects, fields, arguments, etc.). In addition, key arguments/values (e.g., “Covid-19”) from the utterance query missed by the pointer network are copied and populated into the sequence of the final GraphQL statement (Figs. 7, 8). The following token copying probabilities ${p}_{\text{copy}}$ are transformed based on positional probabilities, where $m$ is a candidate for all “translations” of all source inputs in an instance.

24 $$p_{copy} = p\left( {y_{t} \left| {y < t,x,m} \right.} \right)$$

The final probability distribution final can be obtained by linear interpolation of $copy$ and $gen$ (generation probability of the pointer network):

25 $$\mathrm{\alpha }=\left(1-{\upbeta }_{t}\right)\times {p}_{\text{gen}}$$

26 $$p_{final} \left( {y_{t} \left| {y < t,x,m} \right.} \right) = \beta_{t} \times p_{copy} + \alpha$$

${\upbeta }_{t}$ is the dynamic weight of the $t$-th step, which can be characterized as:

27 $${\upbeta }_{t}=Sigmoid\left({W}_{{s}_{t}}^{\mathrm{^{\prime}}}\right)$$

## Experiment

**Fig. 9:**
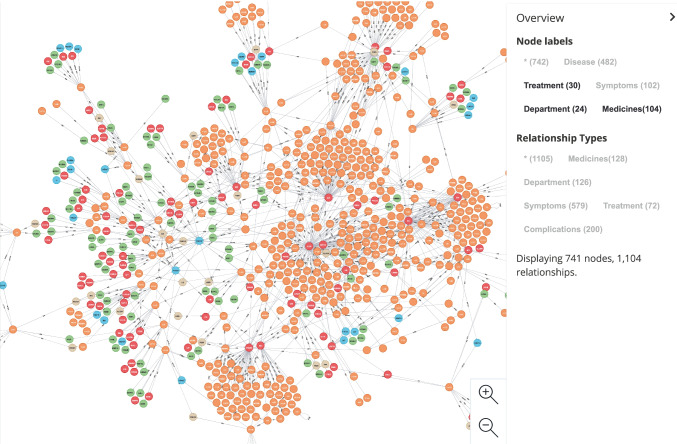
A demo screenshot of medical encyclopaedia knowledge graph for question and answering system

We use a large online medical question-and-answer community (39.net) in Chinese as the data source. First, we extract key information from these question-and-answer data through named entity recognition and relationship extraction models. And through data processing, including disambiguation and entity linking, a medical knowledge graph was constructed (Fig. 9). Based on these data, the content of the question and answer is highly abstract. The key information in the question-and-answer content is extracted, and these entities are connected in the form of a network to form a larger-scale knowledge network. These knowledge networks will serve as an important source of medical information and a basic knowledge base for the question-and-answer system and use the dialogue system as a search source based on this. Therefore, our Text-to-GraphQL method will serve as the upper-level application of the graph database, allowing users to translate natural language into logical expressions (i.e., graph query statements) through the middle-ware.

The experiment of the research will use a large amount of real medical question and answer data collected from 39.net as a dataset for annotation and translation and use this to construct a knowledge Graph in the medical field. The dialogue system part is based on the knowledge graph to construct a question-and-answer system combining regular expressions and deep learning models. Therefore, the core part of the system is the module that transforms natural language into logical formal language through Text-to-GraphQL tasks. In addition, Neo4j GraphQL Library (with Cypher) is one of the necessary components. Therefore, we will verify the effect of our proposed method in this module from multiple criterias. The evaluation indicators are mainly “question matching accuracy” (i.e., the exact set matching overall score questions) and “accuracy of interaction matching” (i.e., the exact set matching overall score interactions).

In addition, since the research field on Text2GraphQL is still blank, there is a lack of available test data. But in the Text2SQL task, there is a lot of available training and test data. Therefore, in this research, we also use the ready-made dataset in the Text2SQL task to test the generalization of the proposed model. This means that the work we need to handle includes the steps of Text2SQL dataset processing and format conversion, supplementary data annotation and manual inspection. The details of these steps will be elaborated on in the following sections.

### Experiment Datasets

#### Spider Dataset

Currently, there are Text2SQL corpora for different fields, and the datasets can be used to transform into GraphQL query datasets. As one of the most comprehensive Text2SQL datasets, Spider contains complex queries and SQL clauses. It contains a broad domain spanning 200 databases. Make it possible to test the generalization of models across different schemas and domains after being converted to GraphQL format. A new semantic parsing task is defined, in which there is no overlap between the queries and the databases between the training and evaluation datasets. This allows us to confirm the generalization across prompts and databases not included in the training dataset.

#### Other Datasets used to Test the Effect of the Model

Due to the lack of available Text2GraphQL, test datasets for comparative experiments are to verify the performance of the proposed model. Therefore, in addition to providing our annotation dataset based on the real knowledge graph, we also consider increasing our test data by converting the existing Text2SQL datasets. These datasets include: Spider 1.0 (Yu et al., 2018c), ATIS (Dahl et al., 1994) and GeoQuery (Zelle & Mooney, 1996).

#### Approach to Convert SQL to GraphQL Format

Text2SQL (e.g., Spider) provided most of the schemas to its databases, along with a dump of the contents of the databases as an SQLite dump. In addition, Hasura provides the best GraphQL API currently available (“Hasura is an open-source engine that connects to databases and microservices and generates a production-ready GraphQL backend automatically.”). Since Hasura depends on PostgreSQL databases. The SQLite dumps were converted to Postgres Databases through the use of PGLoader (a data loading tool for PostgreSQL). Each database dump can be mapped to a PostgreSQL database through the use of a script, with a few manual edits to the raw dump files. Hasura could then generate a GraphQL schema based on the PostgreSQL schema and database. Subsequently, we confirm the relations, names, and types in the schemas. Finally, we manually verify the metadata of the databases and make some corrections to the schema and values.

Converting SQL to GraphQL through the use of a processing script once the databases and schemas were accessible in a Hasura GraphQL endpoint. Most SQL clauses could be converted using a SQL Abstract Syntax Tree (AST) to GraphQL AST strategy (SQL ASTs include metadata about the clauses, the tables, and the columns in a database). The scripts process involved recursive graph searches in matching GraphQL types and names to SQL tables and columns. Once a GraphQL AST was formed, the AST could be encoded as a GraphQL query in string form. Some Spider queries could not be transferred to Hasura GraphQL queries, because of the limitations of Hasura. “GROUPBY” clauses are not implemented in Hasura and therefore could not be transferred without manually modifying the schemas and queries. Therefore, in this part, we can only use manual correction to realize it.

## Results and Analysis

We are converting Spider 1.0, ATIS, and GeoQuery datasets from SQL format to GraphQL format by the above method. Hence, we turn it into a Text-to-GraphQL task. However, because Hasura is used to convert from SQL query to GraphQL format, this is purely a semantic format conversion rather than converting the corresponding SQL database to Graph database. Hence, in the experimental part of the task of converting Text2SQL to Text2GraphQL, we mainly focus on the accuracy performance of the proposed model on the development and test sets, rather than its actual execution effect on the graph database. Therefore, the test results here are the performance of the model on each converted dataset.

**Fig. 10:**
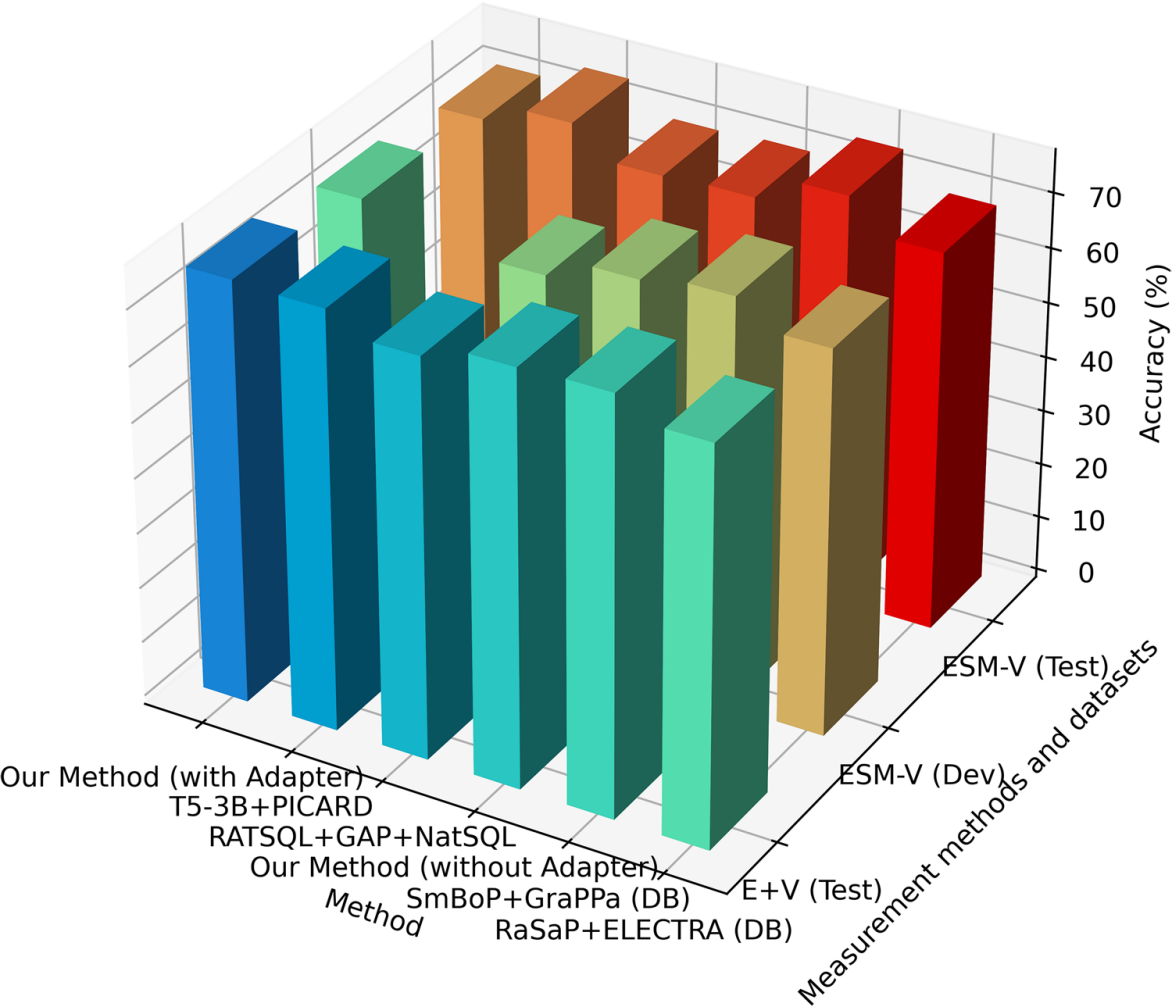
Visualization of comparative models' performance on Spider 1.0 dataset converted to GraphQL format (Top 6)

**Fig. 11:**
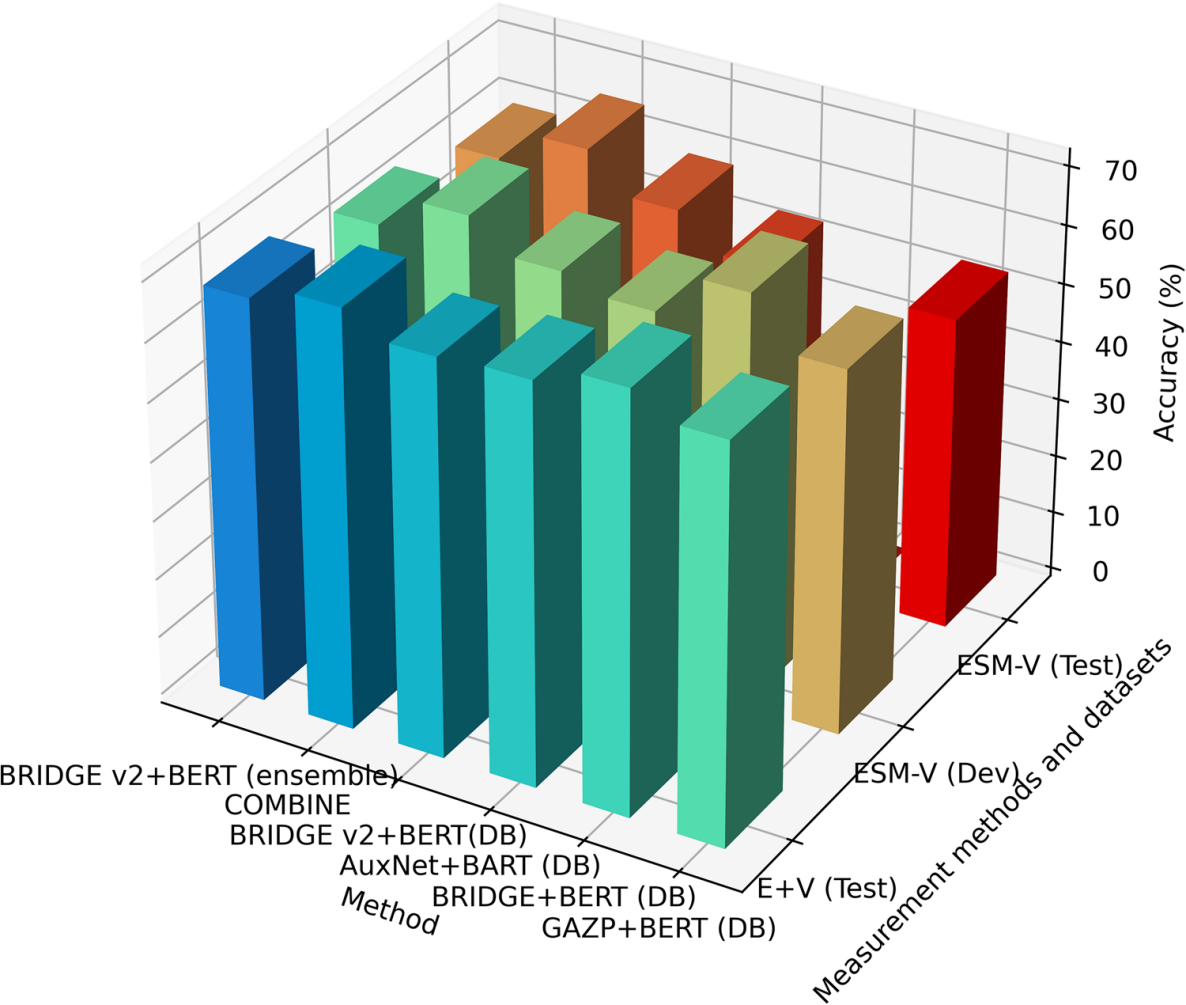
Visualization of comparative models' performance on Spider 1.0 dataset converted to GraphQL format (bottom 6)

According to the above-mentioned conversion methods, we converted the Spider 1.0 data set to GraphQL format. The results of our model and other compared models on this data set are shown in Table 1. It can be found from the table that our proposed model has the best performance (76.2%, 75.8% and 72.3%) respectively on the test set of “Execution with Values”, development set and the test set of “Exact Set Match Accuracy” compared with the current main Text-to-SQL models. This has also been verified in ablation experiments. In the experiment of the model “without Adapter”, the corresponding sets in the above dataset of Spider 1.0 obtained 72.5%, 74.2% and 69.8%, respectively. This has certain advantages over the current main Text2SQL methods (Table 1, Fig. 10, 11).

**Table 1:** Performance of our method on Spider 1.0 dataset (Converted to GraphQL)

Model	Execution with Values (E + V)	Exact Set Match without Values (ESM-V)	
Set of Data	Test	Dev	Test
Our Method (with Adapter)	**76.2**	**75.8**	**72.3**
T5-3B + PICARD (DB content used) (Scholak et al., 2021)	75.1	75.5	71.9
RATSQL + GAP + NatSQL (DB content used) (Gan et al., 2021)	73.3	-	68.7
Our Method (without Adapter)	72.5	74.2	69.8
SmBoP + GraPPa (DB content used) (Rubin & Berant, 2021)	71.1	74.7	69.5
RaSaP + ELECTRA (DB content used) (Huang et al., 2021)	70.0	74.7	69.0
BRIDGE v2 + BERT (ensemble) (DB content used) (Lin et al., 2020)	68.3	71.1	67.5
COMBINE (DB content used) (Mellah et al., 2021)	68.2	71.4	67.7
BRIDGE v2 + BERT (DB content used) (Lin et al., 2020)	64.3	70.0	65.0
AuxNet + BART (DB content used)	62.6	70.0	61.9
BRIDGE + BERT (DB content used) (Lin et al., 2020)	59.9	65.5	59.2
GAZP + BERT (DB content used) (Zhong et al., 2020)	53.5	-	53.3

**Fig. 12:**
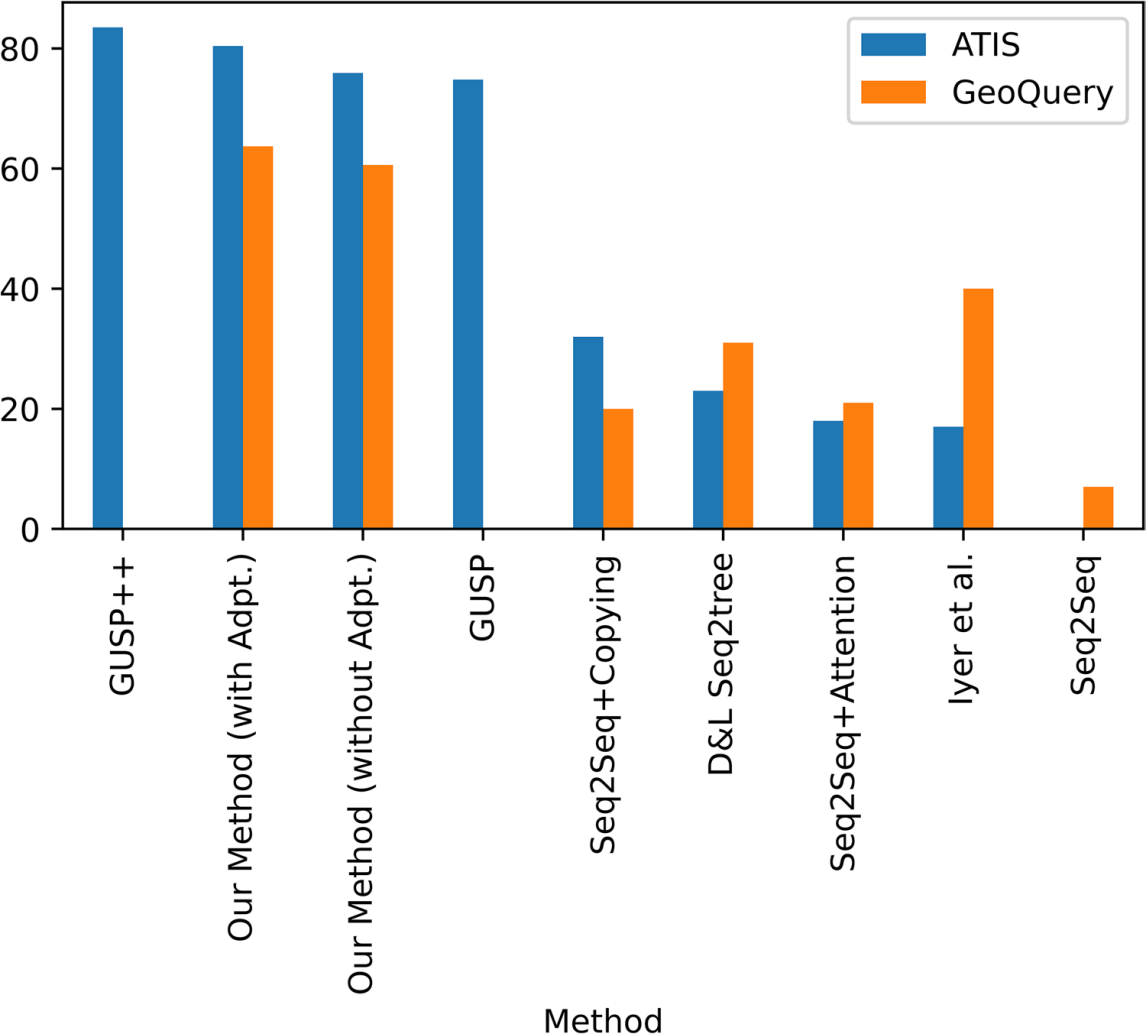
Visualization of comparative models' performance on ATIS and GeoQuery datasets converted to GraphQL format

Compared with several previous methods that combine the language model and database content (Huang et al., 2021; Lin et al., 2020; Zhong et al., 2020), our proposed method can improve the overall performance (83.5% and 63.7%) by introducing a priori knowledge of schemas and the corresponding utterance. Similarly, the overall performance of the proposed method on ATIS and GeoQuery datasets is generally better than that of Seq2Seq type methods and their variants (Finegan-Dollak et al., 2018; Iyer et al., 2017; Poon, 2013). At the same time, according to the ablation experiments on these two datasets, the model “without Adapter” can also achieve 75.9% and 60.6% accuracy, which is at the forefront of all the current major comparable methods (Table 2, Fig. 12). Therefore, the proposed method has been verified for generalization on multiple datasets.

**Table 2:** Performance of the proposed method on ATIS (Dahl et al., 1994) and GeoQuery (Zelle & Mooney, 1996) datasets (Converted to GraphQL)

Model|Dataset	ATIS	GeoQuery
GUSP + + (Poon, 2013)	**83.5**	-
Our Method (with Adapter)	80.4	**63.7**
Our Method (without Adapter)	75.9	60.6
GUSP (Poon, 2013)	74.8	-
Seq2Seq + Copying (Finegan-Dollak et al., 2018)	32	20
D&L Seq2tree (Finegan-Dollak et al., 2018)	23	31
Seq2Seq + Attention (Finegan-Dollak et al., 2018)	18	21
Iyer et al. (Iyer et al., 2017)	17	40
Seq2Seq (Finegan-Dollak et al., 2018)	0	7

**Fig. 13:**
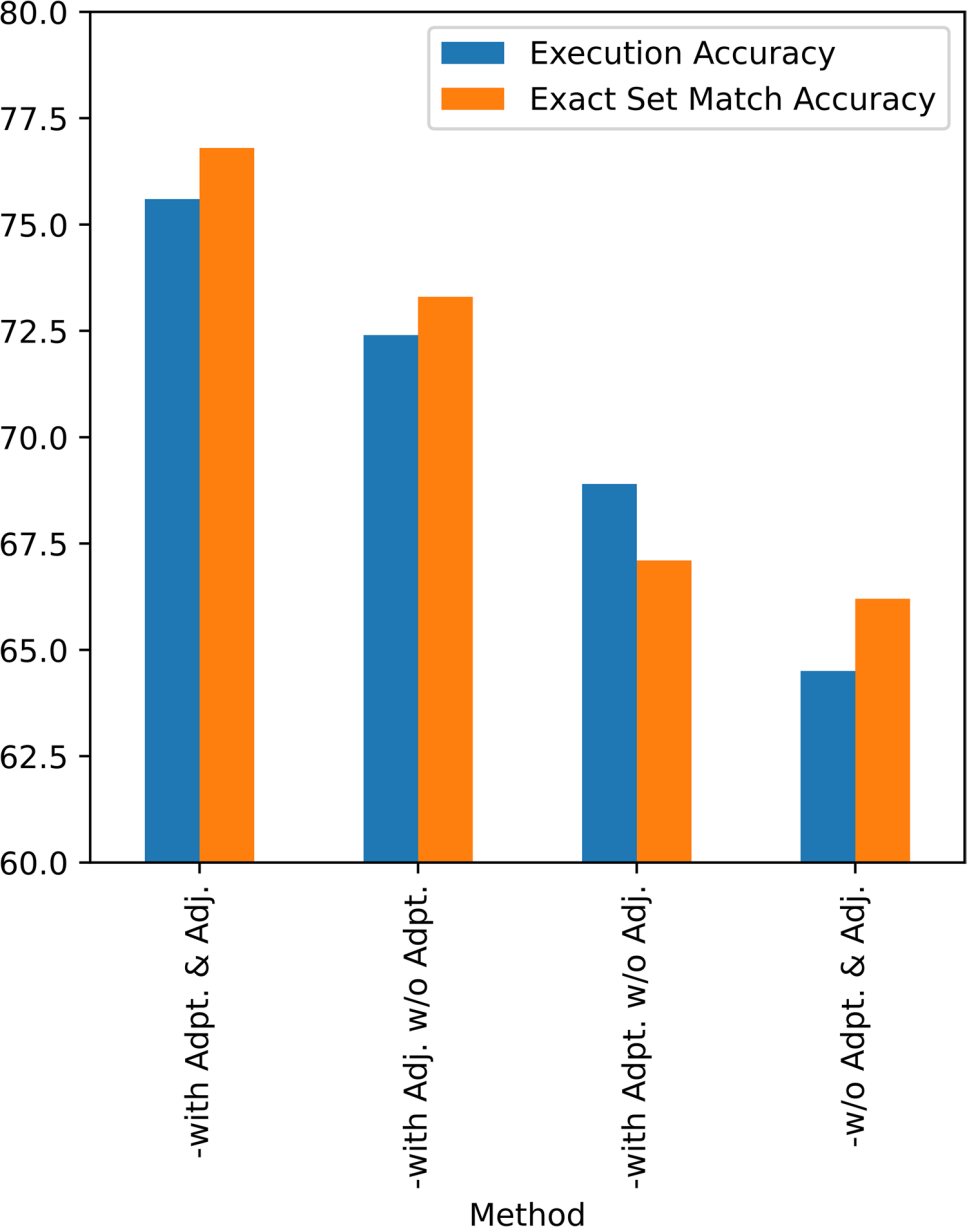
Visualization of performance of the proposed model with different mechanisms on 39.net medical Text2GraphQL dataset

The results on the 39.net medical Q&A dataset show that the method of the “with Adapter and Adjuster” mechanism is better than other methods in both “Execution Accuracy” and “Exact Set Match Accuracy”. From the comparison of the effects of the “with Adjuster without Adapter” and “with Adapter without Adjuster” mechanisms, the Adjuster improves the overall output of the model even more. Finally, the model leaves the Adapter and Adjuster mechanism, which is 11.1% and 10.6% respectively, lower in performance than the full version. Therefore, these results can demonstrate the influence and extent of the Adapter and Adjuster mechanism on the overall performance of the model (Table 3, Fig. 13).

**Table 3:** Performance of our proposed method on the 39.net medical Q&A Text2GraphQL dataset

Model	Execution Accuracy	Exact Set Match Accuracy
Proposed Model-with Adapter and Adjuster	75.6	76.8
-with Adjuster without Adapter	72.4	73.3
-with Adapter without Adjuster	68.9	67.1
-without Adapter and Adjuster	64.5	66.2

## Conclusions

A large part of the medical consultation system based on knowledge graphs relies on the realization of the function of graph database retrieval. And this task can be realized by transforming the natural language query into the logical form of the graph query statement. In this research, we propose an encoder-decoder pipeline composed of a language model with a “schema-utterance” knowledge introduction mechanism and a Pointer Network with complex computing mechanisms. Among them, we have inserted the “Adapter” layer pre-trained with “Schema-Utterance” knowledge in the language model, and the entire language model can be regarded as a smarter encoder of the whole pipeline. This allows the encoder to use the rich a priori natural language knowledge in the language model, as well as the matching knowledge of the schema corresponding to different utterances, to perform a more accurate logical formal translation of the input natural language query. The improved Pointer Network, as a decoder, can use the Attention mechanism to calculate the weight of the content vector on the logical form sequence input by the encoder and use the guide mechanism based on the GraphQL schema dictionary to restrict the distribution of weights in the corresponding reasonable range. Finally, through the calculation of weights and probability distributions, the token output at each step is determined, thereby forming the output of the complete GraphQL statement sequence. This research also verified the practical ability of the model by testing the execution results of the 39.net medical Q&A knowledge graph and the derived dataset, which includes natural language questions and corresponding GraphQL queries. And through the comparative experiments of Spider 1.0, ATIS, and GeoQuery, the effect and generalization ability of the proposed method have been proved to a certain extent. As the first public work of Text-to-GraphQL, this work provides some enlightenment for the development of this field.

## Limitations and Future Works

In the next step, Life-Long Learning will be used to further realize the extractor of information entities, relationships, and semantic structure of sustainable learning, to better realize the expansion of the continuous increment knowledge graph and provide more knowledge accumulation for the question-and-answer system. Also, specialized medical domain language models (Lee et al., 2020; Ni et al., 2021a; Zhu et al., 2019, p. 2) will be used for higher-performing Text2GraphQL models. Including autonomous methods such as deep reinforcement learning (Li et al., 2019a, 2020; Yu et al., 2018a, 2018b), will be combined with crowdsourcing and other methods, to be used as schemas and their various combinations to match more corresponding utterances.

And limited by the lack of available graph database as execution support for the data set converted by Text2SQL, the proposed model cannot be verified on Spider 1.0 in terms of execution accuracy. Similarly, since there are no other methods available for Text2GraphQL tasks, the proposed model cannot be compared to the 39.net medical question and answer data set. This will also be the key exploration direction in the future.
